# Bio-Adhesive Lignin-Reinforced Epoxy Acrylate (EA)-Based Composite as a DLP 3D Printing Material

**DOI:** 10.3390/polym17212833

**Published:** 2025-10-23

**Authors:** Jeonghong Ha, Jong Wan Ko

**Affiliations:** 3D Printing Manufacturing Process Center, Smart Forming Process Group, Korea Institute of Industrial Technology (KITECH), Ulsan 44776, Republic of Korea; jhjh@kitech.re.kr

**Keywords:** digital light processing, bio-adhesive lignin, reinforced plastics, tensile strength, hardness

## Abstract

Digital light processing (DLP) 3D printing is a powerful additive manufacturing technique but is limited by the relatively low mechanical strength of cured neat resin parts. In this study, a renewable bio-adhesive lignin was introduced as a reinforcing filler into a bisphenol A-type epoxy acrylate (EA) photocurable resin to enhance the mechanical performance of DLP-printed components. Lignin was incorporated at low concentrations (0–0.5 wt%), and three dispersion methods—magnetic stirring, planetary mixing, and ultrasonication—were compared to optimize the filler distribution. Cure depth tests and optical microscopy confirmed that ultrasonication (40 kHz, 5 h) achieved the most homogeneous dispersion, yielding a cure depth nearly matching that of the neat resin. DLP printing of tensile specimens demonstrated that as little as 0.025 wt% lignin increased tensile strength by ~39% (from 44.9 MPa to 62.2 MPa) compared to the neat resin, while maintaining similar elongation at break. Surface hardness also improved by over 40% at this optimal lignin content. However, higher lignin loadings (≥0.05 wt%) led to particle agglomeration, resulting in diminished mechanical gains and impaired printability (e.g., distortion and incomplete curing at 1 wt%). Fractographic analysis of broken specimens revealed that well-dispersed lignin particles act to deflect and hinder crack propagation, thereby enhancing fracture resistance. Overall, this work demonstrates a simple and sustainable approach to reinforce DLP 3D-printed polymers using biopolymer lignin, achieving significant improvements in mechanical properties while highlighting the value of bio-derived additives for advanced photopolymer 3D printing applications.

## 1. Introduction

Additive manufacturing (AM), also known as 3D printing, has emerged as a transformative manufacturing technique that enables the fabrication of complex three-dimensional structures directly from digital designs [1,2]. Owing to its high design flexibility and tool-free production, AM has found applications across diverse industries, including medical, aerospace, and electronics [3]. Polymer-based 3D printing technologies have attracted significant attention due to their ability to fabricate intricate three-dimensional structure that are difficult to achieve using conventional manufacturing methods [4]. These polymer 3D printing techniques are generally classified into vat photopolymerization (VPP) [5,6], material extrusion (ME) [7], powder bed fusion (PBF) [8,9], and material jetting (MJ) [10,11]. In VPP, a liquid photocurable resin is selectively cured layer by layer through light irradiation [5,6]. ME, one of the most widely used technologies, involves melting a thermoplastic filament and extruding it layer by layer [7]. PBF works by spreading ultra-thin layers of polymer powder and selectively fusing or melting targeted areas with a high-powered laser or electron beam [8,9]. MJ directly jets build and support material droplets through a print head, depositing them layer by layer as photocurable inks [10,11].

Among polymer AM methods, digital light processing (DLP) 3D printing, which is a VPP technique, has attracted considerable attention due to its advantage such as high lateral resolution, rapid printing speed, and uniform surface quality of printed parts [12,13]. Beyond prototyping, DLP has been increasingly applied to functional devices, biomedical scaffolds, and high-performance structural components, reflecting its rapid industrial expansion. However, DLP 3D printing has critical limitations stemming from its reliance on photocurable resins and photoinitiators. The cured parts produced from a pure (neat) resin often exhibit relatively low mechanical properties, which can limit their suitability for demanding industrial applications [14,15]. To overcome these limitations of DLP, various strategies have been explored, including resin chemistry modification (e.g., incorporation of toughening agents, elastomeric monomers, or dynamic covalent bonds), interfacial and interlayer enhancement techniques (e.g., molecular diffusion, surface modification) [16,17], post-processing approaches (e.g., post-curing and thermal annealing) [18], optimization of printing parameters such as layer thickness and exposure conditions [19], and the design of composite materials through the incorporation of fillers [20,21]. These strategies highlight that both material design and process control are essential to bridging the performance gap between DLP-printed polymers and conventionally manufactured engineering plastics.

The incorporation of fillers into photocurable resins has emerged as a particularly promising route to enhance mechanical properties of the DLP-printed parts [17,22,23]. Senthooran et al. reported that an epoxy acrylate resin reinforced with mica flakes exhibited nearly 85% higher tensile strength compared to the neat matrix [22], while another study showed that 0.5 wt% of graphene additives could yield 38.1% improvement in tensile strength [23]. More recently, bio-derived fillers such as cellulose [24], wood flour [25], and agricultural residues [26] have been introduced as sustainable alternatives, achieving tensile strength increases on the order of 10–25% depending on filler type and dispersion quality. While higher filler loadings generally lead to further improvements in mechanical performance, they are often accompanied by drawbacks such as increased resin viscosity, agglomeration, and excessive light absorption, which can compromise printability and curing efficiency [27,28,29]. Therefore, achieving a balance between mechanical reinforcement and resin formulation, along with process optimization, has become a critical research focus for filler-reinforced DLP-printed parts.

Lignin constitutes approximately 20–35% of woody biomass and is a major component of the lignocellulosic matrix [30]. As a natural polymer, lignin plays a key role in providing the strength and durability to wood, and its bio-adhesive properties also make it attractive for biomedical materials and applications [31]. Moreover, lignin, obtained as a low-cost byproduct of the wood pulping industry, is a renewable resource that can reduce environmental impact when utilized as a new material [32]. Lignin has accordingly attracted increasing attention across various industries, including forestry, chemical and energy sectors [33,34,35]. Recent studies have even explored the optoelectronic properties of lignin, opening new avenues for advanced applications [36,37]. Given its ability to enhance composite strength in other contexts, lignin is expected to improve the mechanical properties of DLP-printed polymer parts. The motivation for utilizing lignin in this study originates from its natural role as a reinforcing component in wood and its potential to impart similar reinforcement in synthetic polymer matrices. Recently, several studies have explored the incorporation of lignin into photocurable resins for vat photopolymerization processes such as stereolithography (SLA) and digital light processing (DLP). Arias-Ferreiro et al. investigated unmodified lignin as a filler in an acrylic-based photocurable resin and reported that additions up to 4 wt% improved tensile and hardness properties but caused a significant reduction in curing depth and print quality at concentrations above 1 wt% [38]. Similarly, Nguyen et al. examined lignin-containing acrylate and epoxy systems with lignin contents typically in the range of 1–5 wt%, observing moderate reinforcement but also viscosity increase, phase separation, and light attenuation that limit the DLP printability of the resin [39]. These previous studies demonstrated the potential of lignin as a bio-based reinforcing filler but also revealed inherent limitations associated with particle aggregation, increased viscosity, and reduced curing depth at higher loadings, which can ultimately degrade printability and mechanical uniformity in DLP systems.

**Figure 1:**
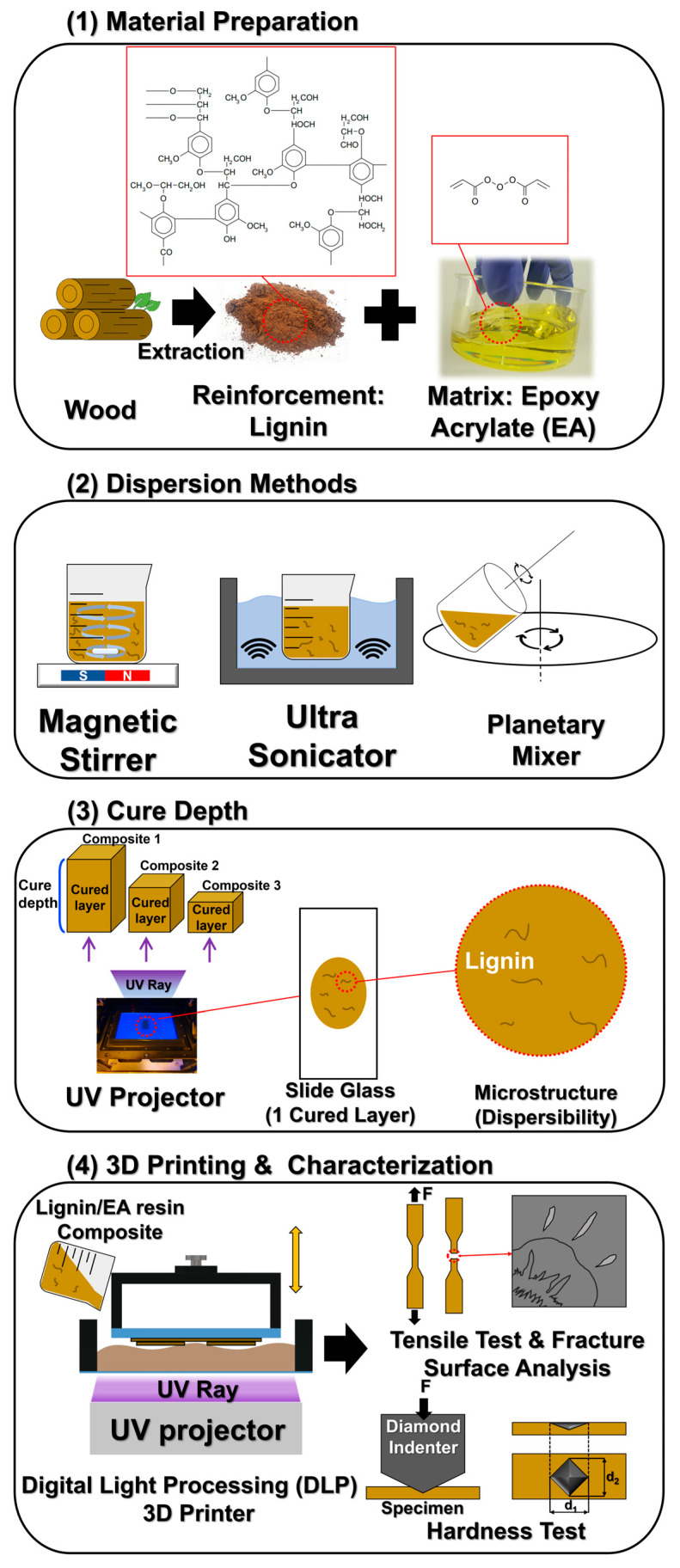
Schematic illustration of the overall process: (1) material preparation of lignin and EA resin, (2) dispersion methods (magnetic stirring, ultrasonication, planetary mixing), (3) cure depth measurement mechanism, and (4) DLP 3D printing procedure and characterization of printed specimens.

In this work, bio-adhesive lignin was incorporated as a reinforcing filler into an epoxy-acrylate-based resin for DLP 3D printing, which aimed to improve the inherently poor mechanical properties of neat photopolymer resin. Figure 1 illustrates the overall process of the study, including (1) material preparation of lignin and the base EA resin, (2) dispersion of lignin using three different methods (magnetic stirring, ultrasonication, and planetary mixing), (3) the cure depth test used to evaluate resin printability, and (4) the DLP 3D printing of sample specimens followed by material characterization. Unlike previous studies that employed relatively high lignin loadings (typically 1–5 wt%) or used a single dispersion method, this work systematically compared multiple mixing approaches and identified ultrasonication as the optimal technique for achieving homogeneous lignin dispersion without sacrificing curing efficiency. To identify the optimal dispersion conditions, we carefully examined the curing depth of the resin and the microstructural dispersion of lignin under each mixing method. Using the optimized dispersion method, DLP 3D-printed specimens were fabricated with varying lignin contents (0–0.5 wt%) to assess improvements in mechanical properties. The enhancements in mechanical performance of the printed samples were analyzed through tensile testing and hardness measurements. Overall, this study demonstrates the feasibility of leveraging a small amount of lignin as a natural filler to significantly enhance the mechanical performance of DLP 3D-printed parts, while also contributing to sustainable material use and resource recycling.

## 2. Materials and Methods

### 2.1. Material Preparation and 3D Printing

The base photocurable resin used for DLP 3D printing was a bisphenol A-type epoxy acrylate (EA; H200, Laonix Co., Ltd., Ulsan, Republic of Korea). Prior to use, the EA resin was degassed in a vacuum desiccator for 24 h to remove entrapped air bubbles. The reinforcing filler was a lignin powder (Lignum Co., Ltd., Daejeon, Republic of Korea), used as received without further treatment. Lignin/EA resin composite mixtures were prepared with various lignin loadings (0–0.5 wt%) to investigate the reinforcement effect of lignin on the polymer matrix.

To optimize dispersion, three different mixing methods (magnetic stirring, planetary mixing, and ultrasonication) were employed. For magnetic stirring, a lab-scale stirrer (MSH-20D, DAIHAN Scientific, Wonju, Republic of Korea) was used at 800 rpm for varying durations of 2, 4, 6, 8, and 24 h to evaluate the dispersion of lignin in the EA resin over time. For planetary mixing, a planetary mixer (KK-250SE, KURABO Co., Ltd., Osaka, Japan) was operated at revolution speeds of approximately 640–1700 rpm (setting levels 1–10), with the rotation speed set to 0.0–1.0 times the revolution speed (levels 0–9). The composite was mixed in the planetary mixer for durations ranging from 220 s up to 1100 s, depending on the target dispersion condition. In the shortest case (220 s total), a three-step mixing sequence was applied: Step 1—40 s at revolution level 3/rotation level 3; Step 2—120 s at revolution level 6/rotation level 6; Step 3—60 s at revolution level 7/rotation level 6. This stepwise mixing protocol was designed to ensure sufficient dispersion of lignin while simultaneously de-aerating the resin. For ultrasonication, an ultrasonic bath (POWERSONIC 610, Hwashin Tech, Seoul, Republic of Korea) operating at 40 kHz was used to disperse lignin in the resin for durations ranging from 1 to 6 h.

Tensile test specimens (dog-bone shape, ASTM D638 Type V [40]) were designed in CAD and exported as STL files. The specimens were fabricated on a DLP 3D printer (EDGE 200, Laonix Co., Ltd., Ulsan, Republic of Korea) with a resolution of 2560 × 1600 pixels (pixel size ~70 µm). The printing parameters were as follows: light source wavelength of 405 nm, light intensity of 10 mW/cm^2^, layer exposure time of 10 s, and layer thickness (coating thickness) of 200 µm. After printing, all specimens were post-cured under a 405 nm UV lamp for 4 h to ensure full cure of the resin. For the printability tests, tensile specimens were printed using neat EA resin and lignin/EA resin composites containing 0.025 wt% and 1 wt% lignin (representing low and high filler loadings). For the mechanical property tests, specimens were printed using neat resin and composites with lignin contents of 0.01, 0.025, 0.05, 0.125, 0.25, and 0.5 wt%. These ranges were chosen based on preliminary findings to cover the optimal filler amount and beyond.

### 2.2. Characterization and Analysis

To evaluate the effectiveness of each mixing method for dispersing lignin and its impact on resin printability, cure depth measurements were conducted on the neat resin and on composite resins containing 2 wt% lignin (a relatively high concentration chosen to accentuate differences in dispersion). A fixed volume of each resin sample (1 mL) was dispensed onto a microscope slide and exposed to 405 nm UV light (10 mW/cm^2^) for 10 s (mimicking one layer exposure in the DLP printer). The uncured resin was then rinsed away with ethanol, leaving a cured circular disk. The thickness (cure depth) of this cured layer was measured using a digital vernier caliper (Mitutoyo CD-20APX, Kawasaki, Japan). The caliper has a resolution of 0.01 mm, and the cure depth was measured three times for each sample to obtain the mean value and standard deviation. For each mixing method (magnetic stirring, ultrasonication, planetary mixing), cure depth was measured at various mixing times to determine how dispersion quality affected the resin’s curing behavior. An optical microscope (RH-2000, Hirox Co., Tokyo, Japan) was also used to observe the single cured layer on the slide, in order to visually assess the dispersibility of lignin (e.g., presence of visible particles or agglomerates) and thereby help identify the optimal mixing conditions.

The printability and dimensional accuracy of the 3D printed composites were evaluated by comparing the printed specimen dimensions to the original design. Nine specific measurement points on the printed tensile specimens were selected (marked “a” through “i” on the CAD model), and the dimensional deviation at each point was calculated as a percentage difference from the CAD model dimension. By examining these deviations for the neat resin vs. composite samples, we quantified how the addition of lignin (at 0.025 wt% or 1 wt%) influenced the accuracy and reliability of the printing process.

For mechanical testing, tensile tests were performed using a universal testing machine (Quasar 50, Galdabini, Cardano al Campo, Italy) with a 10 kN load cell. Tests followed ASTM D638 Type V standards. The crosshead speed was set to 1.5 mm/min, and each specimen was pulled until failure to obtain stress–strain data. The ultimate tensile strength and elongation at break were recorded for each sample. In addition, hardness tests were carried out on the printed samples using a micro-Vickers hardness tester (MMT-X, Matsuzawa, Akita, Japan). A load of 1000 gf was applied for 10 s for each indentation, and the Vickers hardness number (VHN) was measured. Multiple indents were taken on each sample to ensure repeatability, and the average hardness was reported.

## 3. Results and Discussion

### 3.1. Dispersion Optimization

**Figure 2:**
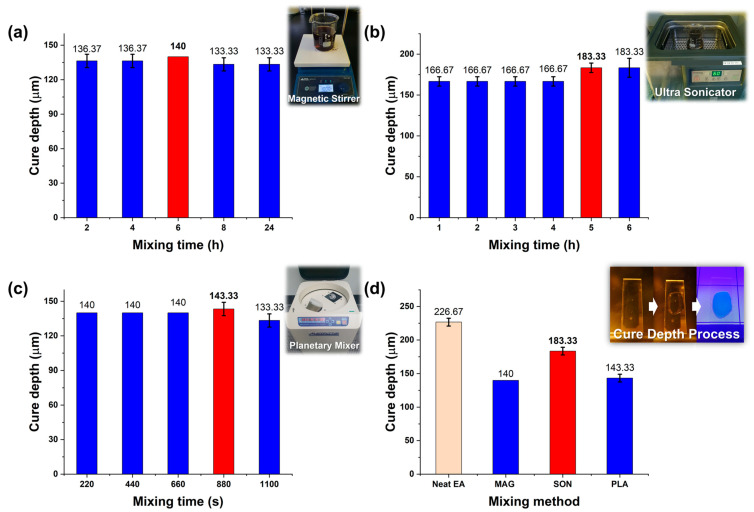
Cure depth of 2 wt% lignin/EA resin composites under different dispersion conditions. Cure depth vs. mixing time for (**a**) magnetic stirring (800 rpm, 2–24 h), (**b**) ultrasonication (40 kHz, 1–6 h), and (**c**) planetary mixing (220–1100 s). (**d**) Maximum achieved cure depth for each method (at optimal mixing time) compared to the cure depth of neat EA resin, including a schematic representation of the cure depth test. Each data point represents the mean value and standard deviation obtained from three repeated measurements using a digital vernier caliper (Mitutoyo CD-20APX, resolution: 0.01 mm). In some cases, the standard deviation appeared as zero because the variation among the measured values was smaller than the measurement resolution. Red bars indicate the best (maximum) cure depth condition in each case.

To determine the most effective method for dispersing lignin in the EA resin, cure depth measurements were performed for each mixing method to evaluate how dispersion quality influences the photopolymerization behavior. Figure 2 summarizes the cure depth results for 2 wt% lignin/EA resin samples under various mixing durations using (a) magnetic stirring, (b) ultrasonication, and (c) planetary mixing, as well as (d) a comparison of the optimal condition from each method against the cure depth of neat resin. As shown in Figure 2a–c, the cure depth of the composite resin varied with mixing time for each method. Both magnetic stirring and planetary mixing exhibited a trend where the cure depth initially increased but then decreased with excessive mixing time, suggesting that prolonged mixing might lead to re-agglomeration of lignin particles or insufficient additional dispersion benefit once a certain energy input is exceeded. In contrast, ultrasonication yielded a notably higher cure depth overall, reaching a maximum of about 183.3 µm after 5 h of sonication (Figure 2b). Figure 2d highlights the best result from each method: the ultrasonicated sample achieved the greatest cure depth among the three methods, approaching that of the neat resin (~226.7 µm). These results indicate that ultrasonication is the most effective dispersion method for lignin in the EA resin under the tested conditions. Unlike magnetic stirring and planetary mixing, which rely mainly on mechanical shear and bulk fluid motion, the ultrasonicator introduces high-frequency acoustic energy into the resin, generating intense localized micro-cavitation and shear forces. This effect can break apart lignin particle agglomerates and promote a more homogeneous dispersion of the filler, thereby minimizing UV light scattering and absorption by undispersed particle clusters. As a result, even though lignin itself is non-photoreactive, the cure depth of the well-dispersed lignin/resin mixture remains relatively high. This confirms both the good dispersibility of lignin and the compatibility of the lignin–resin system under optimized ultrasonication conditions.

**Figure 3:**
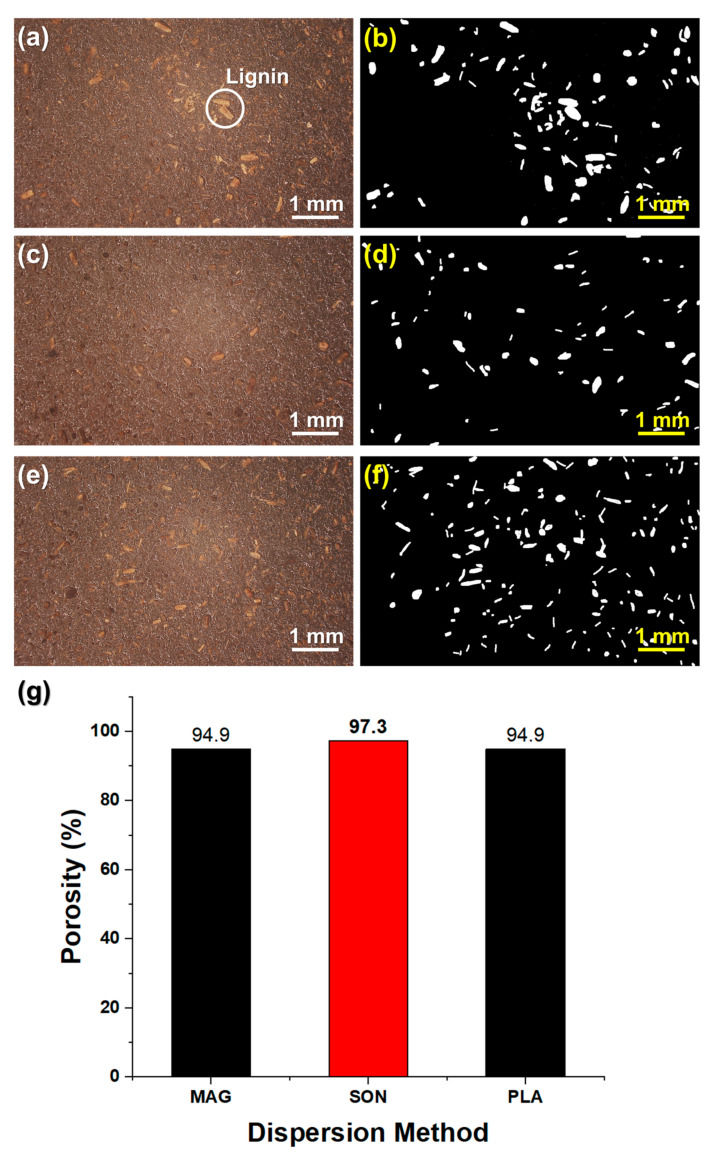
Optical microscopy (OM) images of cured resin layers (2 wt% lignin) after dispersion by different methods: (**a**,**b**) magnetic stirring (800 rpm, 2 h), (**c**,**d**) ultrasonication (40 kHz, 5 h), and (**e**,**f**) planetary mixing (880 s). For each method, the left image (**a**,**c**,**e**) is the OM micrograph showing lignin dispersion, and the right image (**b**,**d**,**f**) is the corresponding binary image used to calculate lignin particle area fraction. (**g**) Measured area fraction (%) of lignin in the composites for each dispersion method, confirming superior dispersion (lower area percentage) with ultrasonication. The red bar indicates the highest porosity (best dispersion) among the tested methods.

To further validate the state of lignin dispersion for each method, optical microscopy (OM) analysis was performed on the cured resin samples (2 wt% lignin) prepared under the optimal mixing duration for each method. The OM images in Figure 3a,c,e reveal noticeable differences in particle visibility and clustering. The samples prepared by magnetic stirring and planetary mixing show clearly visible lignin particles and some localized clusters or agglomerates in the cured resin (Figure 3a and 3e, respectively). In contrast, the sample dispersed by ultrasonication (Figure 3c) exhibits a much more uniform microstructure, with significantly fewer distinct particles observable, indicating a finer dispersion of lignin. To quantify these observations, image analysis was conducted on the micrographs to calculate the area fraction occupied by lignin particles (Figure 3b,d,f show analyzed binary images for each case). As summarized in the graph of Figure 3g, the lignin particle area percentage in the ultrasonicated sample was only ~2.7%, compared to about 5.1% and 5.1% in the magnetically stirred and planetary mixed samples, respectively. This quantitative comparison corroborates the cure depth results, confirming that the ultrasonication method produced a superior dispersion (i.e., fewer and smaller agglomerates) of lignin in the resin. Although the planetary-mixed samples (Figure 3f) seem to exhibit relatively uniform particle distribution at first glance, this apparent homogeneity results from the presence of a larger number of coarse lignin particles that remain undispersed after mechanical shear mixing. These larger residual particles contribute to a visually uniform texture in the binary image but indicate incomplete dispersion. In contrast, ultrasonication effectively breaks down such aggregates into smaller particles, resulting in both a lower area fraction and finer dispersion of lignin throughout the matrix. Therefore, ultrasonication (40 kHz for 5 h) was identified as the optimal dispersion technique and was employed for preparing the composite resins used in subsequent printability and mechanical testing.

### 3.2. Printability and Dimension Reliability

**Figure 4:**
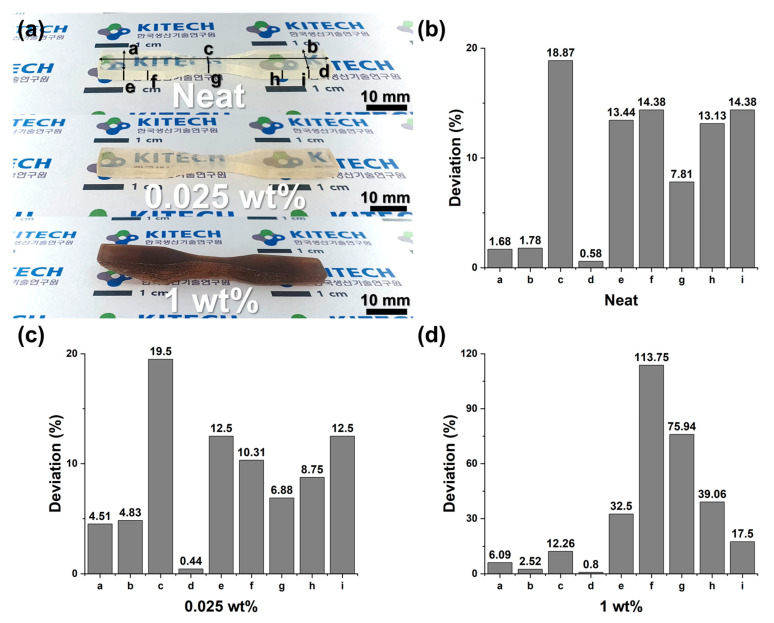
Dimensional accuracy of printed tensile specimens for neat and lignin-filled resins. (**a**) Diagram of the tensile specimen with measurement sections “a”–“i” and photographs of printed samples (neat, 0.025 wt% lignin, 1 wt% lignin) illustrating printability. (**b**–**d**) Dimensional deviation (%) at each section for (**b**) neat resin, (**c**) 0.025 wt% lignin composite, and (**d**) 1 wt% lignin composite, relative to the CAD design. Small deviations in (**b**,**c**) indicate high print fidelity, whereas large deviations in (**d**) reflect print failure at 1 wt% lignin loading.

In the previous section, we established ultrasonication as the most effective method to disperse lignin uniformly in the resin. Using this optimized dispersion technique, we next evaluated the printability and dimensional fidelity of the lignin/EA resin composites. Dog-bone tensile specimens were printed from three resin formulations: neat EA resin, 0.025 wt% lignin/EA composite, and 1 wt% lignin/EA composite. Figure 4a illustrates the measurement scheme: nine critical dimensions (labeled “a” through “i” on the specimen diagram) were compared between the CAD design and the printed samples to calculate dimensional deviations. The percentage deviations for each measured section are presented in Figure 4b (neat resin), Figure 4c (0.025 wt% lignin), and Figure 4d (1 wt% lignin).

For the neat resin and the 0.025 wt% lignin composite, the overall dimensional deviations remained within an acceptable range. Most sections showed very small deviations—as low as ~0.14%—and the maximum deviation observed was about 4–5%, indicating high shape fidelity in the printed parts. In particular, the composite with 0.025 wt% lignin maintained stable curing behavior and consistent dimensions across the various measurement points, very similar to the neat resin. These results suggest that adding a small amount of lignin (0.025 wt%) does not significantly hinder the photopolymerization process or light penetration during layer-by-layer curing. The uniform dispersion achieved via ultrasonication likely ensured that the presence of lignin did not introduce noticeable curing defects or distortion, so the printed part accuracy was preserved.

In contrast, the sample with 1 wt% lignin exhibited severe distortion and even partial structural collapse during the printing process (Figure 4a, bottom). The corresponding dimensional analysis (Figure 4d) shows extremely large deviations at certain sections (for example, section “f” exceeded 100% deviation, indicating the feature did not form correctly). The measurements were inconsistent across the specimen, reflecting significant warping and failure in maintaining the intended geometry. This substantial loss of dimensional accuracy is attributed to the excessive lignin loading which compromised resin homogeneity and curing efficiency. At 1 wt%, even with ultrasonication, the dispersion likely remained incomplete, leading to residual particle clusters. These undispersed lignin clusters can act as physical barriers that scatter or absorb the UV light during printing, preventing proper curing in some regions. As a result, whole layers may under-cure or fail to attach, causing poor interlayer bonding and structural instability. The 1 wt% composite could not be reliably printed because the high lignin filler content (~1 wt%) caused such incomplete curing and weak adhesion that the specimen deformed under its own weight during printing. These findings indicate that there is an upper practical limit to how much lignin filler can be added while still retaining good printability. As shown in Figure 4, the resin containing 1 wt% lignin exhibited noticeably reduced printability and dimensional accuracy, indicating that excessive filler content hinders proper curing and layer formation. Although the image of the 0.5 wt% printed specimen is not shown, the sample was successfully fabricated and used for tensile testing. The specimen exhibited a measurable tensile response with a maximum displacement of approximately 25%, which corresponds to the upper limit of the reliable strain range for the DLP-printed dog-bone geometry. Hence, 0.5 wt% was defined as the practical upper limit at which both printability and structural integrity were still maintained for mechanical property evaluation. Therefore, this study focused on formulations with ≤0.5 wt% lignin, within which consistent print quality and part fidelity were maintained.

### 3.3. Mechanical Properties of 3D Printed Specimens

**Figure 5:**
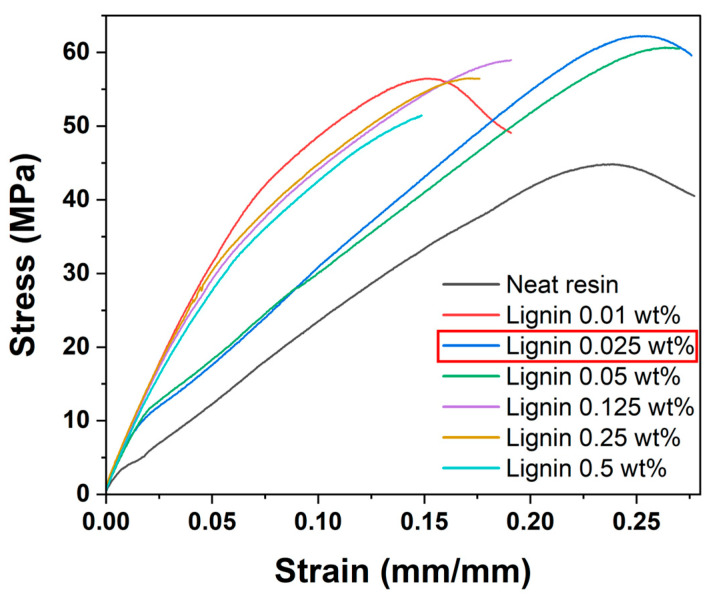
Stress–strain curves of DLP 3D-printed EA resin with different lignin contents (0, 0.01, 0.025, 0.05, 0.125, 0.25, 0.5 wt%). The 0.025 wt% lignin composite shows the highest tensile strength with little loss of elongation, whereas higher lignin contents lead to reduced performance.

After optimizing dispersion and identifying a tolerance of lignin content for printing, we evaluated the mechanical properties of the DLP 3D printed composites. Figure 5 shows representative stress–strain curves for neat EA resin and lignin/EA composites with various lignin contents (0–0.5 wt%). The neat resin displayed a tensile strength of 44.86 MPa and an elongation at break of 0.277 mm/mm. Notably, even a very small addition of lignin produced significant enhancement in mechanical strength. For instance, the composite with 0.025 wt% lignin achieved the highest tensile strength of 62.3 MPa, which is approximately a 39% improvement over the neat resin. Impressively, this strength increase did not come at the expense of ductility. The elongation at break for the 0.025 wt% sample (over 0.276 mm/mm) was essentially comparable to that of the neat resin, indicating that the material retained its deformation capability while becoming stronger.

At the 0.01 wt% lignin content, the tensile strength also increased (to 56.5 MPa), but in this case the elongation at break dropped to 0.191 mm/mm. This suggests that even a small amount of lignin addition can begin to stiffen the matrix and possibly induce brittleness if not optimally dispersed. A minor reinforcement of mechanical strength was achieved at the cost of reduced flexibility, with the 0.01 wt% sample exhibiting less than 70% of the strain observed in the neat resin. As the lignin content was increased beyond 0.025 wt%, both tensile strength and elongation showed a gradual decline. For example, at 0.05 wt% lignin, the tensile strength was 60.7 MPa (still higher than neat resin, but slightly lower than the 0.025 wt% optimum) and the elongation was 0.271 mm/mm (a slight reduction from neat). At 0.125 wt%, the strength further dropped to 59.0 MPa and elongation to 0.191 mm/mm. By 0.5 wt% lignin, the tensile strength (51.4 MPa) had fallen close to the neat resin’s level, and the elongation (0.152 mm/mm, not shown in figure but recorded) was considerably lower. These results indicate that while small amounts of lignin can effectively reinforce the EA resin and improve stress transfer within the polymer network, excessive lignin loadings begin to undermine the composite’s integrity. High filler contents can lead to issues such as particle agglomeration and poor interfacial adhesion between lignin and the polymer, especially if the dispersion is not perfectly maintained at larger scales. The observed decreases in elongation at higher filler contents can be attributed to the following factor: large or poorly bonded lignin clusters act as stress concentrators that initiate early failure under strain. Overall, 0.025 wt% lignin emerged as the optimal content in this study, achieving the best balance between enhancing strength and preserving the material’s ductility. In addition, a slight drop in modulus was observed near 0.02 strain for the 0.025 and 0.05 wt% lignin composites, but not for the higher lignin contents. This transient softening may result from partial interfacial debonding, localized micro-yielding around dispersed lignin particles, or from slight sample slippage at the grips during the early elastic loading stage, which transiently reduce the effective stiffness before the load is redistributed within the matrix [41,42].

**Figure 6:**
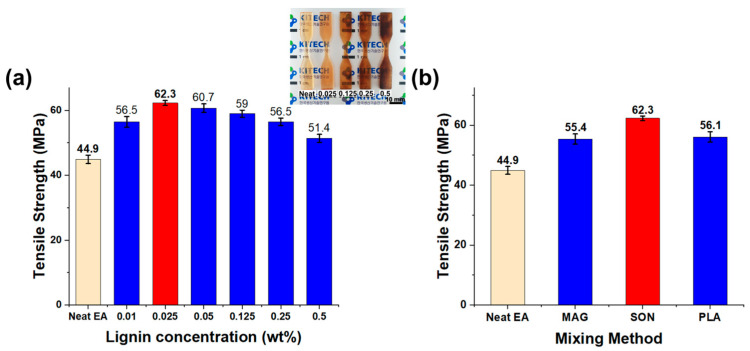
Tensile strength of DLP 3D-printed lignin/EA resin composites. (**a**) Tensile strength vs. lignin content (0–0.5 wt%), showing an optimum at 0.025 wt% and a decline at higher contents. (**b**) Tensile strength of 0.025 wt% lignin composites prepared by different dispersion methods (magnetic stirring, planetary mixing, ultrasonication). Ultrasonication yields the highest strength, correlating with the best dispersion quality. Red bars indicate the best (maximum) tensile strength in each case.

To further examine the effects of lignin content and dispersion method on tensile performance, we compiled the ultimate tensile strength results in Figure 6. Figure 6a compares the tensile strengths of neat resin and composites across the range of lignin contents from 0.01 up to 0.5 wt%. The trend clearly shows the peak at 0.025 wt% and the gradual decline at higher contents, consistent with the earlier discussion. Figure 6b shows on the influence of the dispersion method for the 0.025 wt% lignin composite: it compares the tensile strength of samples prepared by magnetic stirring, planetary mixing, and ultrasonication (all at 0.025 wt% lignin). The ultrasonicated sample achieved the highest tensile strength (62.3 MPa), significantly higher than the strengths of the planetary mixed (56.1 MPa) and magnetically stirred (55.4 MPa) counterparts. This ranking aligns with our cure depth and microscopy findings (Figure 2 and Figure 3), confirming that the superior dispersion attained via ultrasonication directly translates to better mechanical reinforcement. In contrast, the less effective dispersion from simple stirring or planetary mixing leaves more undispersed lignin or micro-voids, thus yielding lower strength improvements even at the same filler content (0.025 wt%).

**Figure 7:**
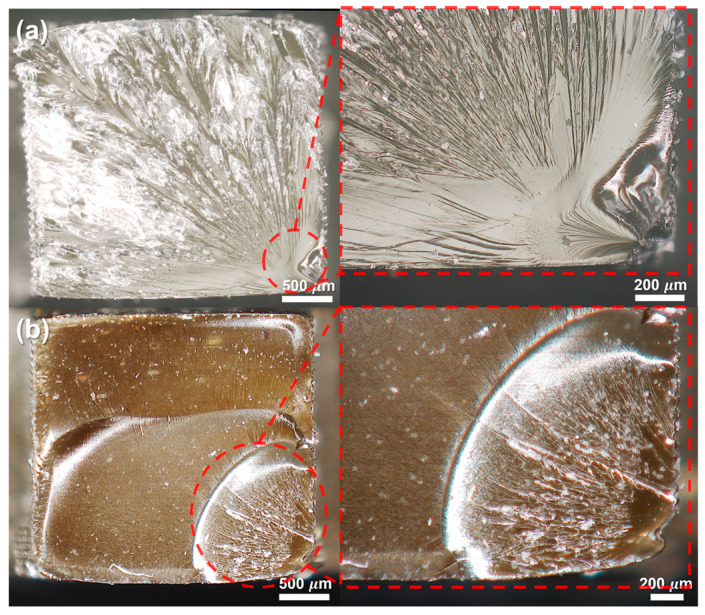
Optical micrographs of fracture surfaces (tensile break cross-sections) of printed specimens: (**a**) neat EA resin and (**b**) 0.025 wt% lignin/EA composite (ultrasonicated). The neat resin shows a relatively smooth, brittle fracture surface, whereas the lignin composite shows a rougher fracture surface with crack deflections and branching, indicating improved toughness due to lignin particles hindering crack propagation.

Fracture surface analysis was conducted to elucidate the reinforcement mechanisms imparted by lignin at the microstructural scope. Figure 7 presents optical microscope images of the fracture surfaces (tensile break cross-sections) for (a) a neat EA resin specimen and (b) a 0.025 wt% lignin/EA composite specimen (ultrasonicated dispersion). The fracture surface of the neat resin (Figure 7a) is relatively smooth and featureless, indicative of a brittle fracture with minimal plastic deformation. In contrast, the fracture surface of the lignin-reinforced composite (Figure 7b) is much rougher and more irregular. The composite shows evidence of crack path tortuosity and micro-scale features such as small stepwise terraced regions and possible branching cracks. This rough fracture morphology suggests that the propagating crack was repeatedly deflected or blunted by obstacles in the microstructure (likely the lignin particles in the EA matrix). In the lignin filled composite, the rigid lignin particles can act as barriers or pinning sites to a growing crack. When a crack front encounters a well-bonded lignin particle, it must either detach the particle, go around it, or initiate a new crack path (branch) deviating from the original direction. Each of these processes consumes additional energy [43]. Consequently, the presence of dispersed lignin makes crack propagation more difficult (the crack can no longer run straight through the material as it did in the neat resin). The need to circumvent or overcome the lignin particles leads to a higher fracture energy absorption, which manifests as a tougher material and a rougher fracture surface. In essence, the lignin particles inhibit crack propagation by increasing the crack path length (through deflection and branching) and by promoting mechanisms like crack bridging or particle pull-out, which further absorb energy. This fracture behavior directly correlates with the improved tensile strength and toughness observed in the mechanical tests of the composite as shown in previous studies [44,45,46]. The fractography thus provides clear visual evidence that even a very small amount of well-dispersed lignin (0.025 wt%) can reinforce the resin matrix by altering and impeding the failure process, resulting in a stronger and more damage-tolerant 3D printed part. The mechanical properties of the neat resin and lignin composites are summarized in Table 1. This enhancement indicates that well-dispersed lignin particles can act as microstructural toughening sites, promoting effective stress transfer and energy dissipation during deformation. These features can contribute to improved resistance to crack propagation and mechanical properties compared with the neat resin.

**Figure 8:**
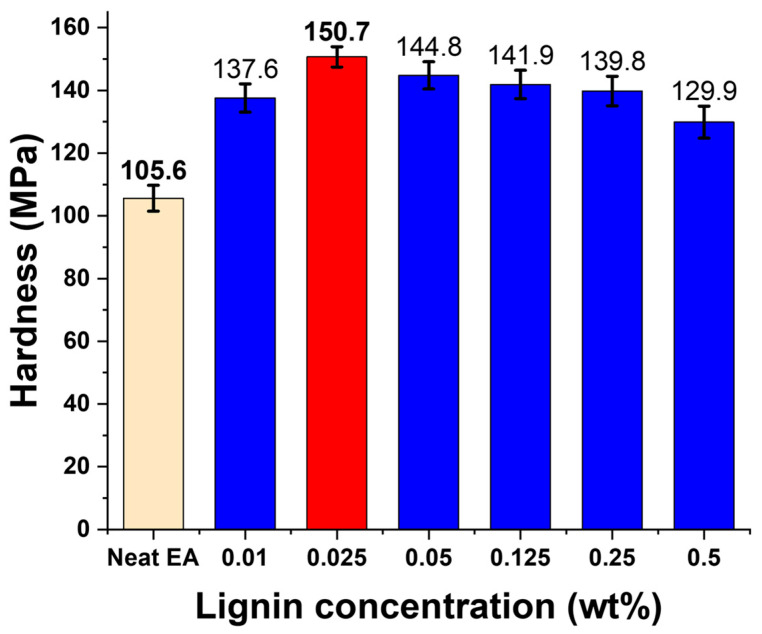
Vickers microhardness of DLP 3D-printed lignin/EA resin composites at different lignin contents (0–0.5 wt%). Hardness increases significantly with lignin addition up to 0.025 wt%, then gradually decreases at higher contents due to dispersion issues, mirroring the trend observed in tensile properties.

In addition to tensile testing, the hardness of the lignin/EA composites was evaluated to assess surface mechanical properties. As shown in Figure 8, the neat EA resin exhibited a Vickers microhardness of 105.57 MPa (this corresponds to the Vickers hardness number converted to an equivalent stress). This relatively low hardness is consistent with the neat resin’s softer, unreinforced polymer network. Upon incorporation of lignin, a substantial enhancement in hardness was observed. At just 0.01 wt% lignin, the hardness increased to 137.62 MPa, and it reached a peak value of 150.66 MPa at 0.025 wt% lignin—which is over a 42% increase compared to the neat sample. This result indicates that a very small amount of lignin not only contributes to bulk mechanical strength (tensile strength) but also significantly improves the material’s resistance to surface indentation. The increase in hardness at 0.025 wt% suggests better load distribution and resistance to local deformation, likely due to the stiff nature of lignin particles and their good adhesion within the matrix, which constrains the polymer chain mobility under the indenter.

## 4. Conclusions

In summary, we have successfully developed a bio-adhesive lignin-reinforced epoxy acrylate composite resin for DLP 3D printing and demonstrated significant improvements in both processability and mechanical performance. A critical outcome of this work is the identification of an optimized dispersion technique—ultrasonication—which enables the uniform distribution of lignin nanoparticles in the photocurable resin without compromising the DLP printing process. By employing ultrasonication (40 kHz, 5 h) prior to printing, even a very low filler loading (around 0.025 wt% lignin) was sufficient to achieve notable reinforcement of the polymer matrix. Under these conditions, the DLP-printed composite parts exhibited a ~39% higher tensile strength and over 40% higher surface hardness compared to parts printed from neat resin, all while preserving the material’s ductility and print fidelity.

The enhanced mechanical properties are attributed to the effective stress transfer and crack-propagation inhibition provided by the well-dispersed lignin particles. Fracture surface analysis confirmed that lignin acts as a microstructural toughening agent, hindering crack initiation and growth through mechanisms such as crack deflection and bridging. Notably, our findings show that more is not always better with respect to filler content: there exists an optimal lignin concentration (around 0.025 wt%) beyond which mechanical benefits taper off due to particle agglomeration and printing difficulties. This work highlights a sustainable and practical strategy for improving the mechanical performance of DLP 3D-printed polymers by using a tiny amount of a renewable bio-filler. The incorporation of lignin not only contributes to stronger and harder printed components but also adds eco-friendly value to the material system by recycling a biomass-derived byproduct. The approach presented here can be extended to other photopolymer systems and fillers, and it opens up new possibilities for creating high-performance, greener materials for advanced additive manufacturing applications.

## Figures and Tables

**Table 1:** Mechanical properties of neat and lignin-reinforced EA resins.

Lignin Concentration [%]	Young’s Modulus [MPa]	Tensile Strength [MPa]	Toughness [MJ/m^3^]
Neat EA	434.0 ± 12.7	44.9 ± 1.3	7.9 ± 0.2
0.01	774.0 ± 22.2	56.5 ± 1.6	7.8 ± 0.2
0.025	650.4 ± 7.8	62.3 ± 0.8	10.6 ± 0.1
0.05	651.3 ± 14.3	60.7 ± 1.3	9.9 ± 0.2
0.125	757.9 ± 14.3	59.0 ± 1.1	7.5 ± 0.1
0.25	753.5 ± 15.3	56.5 ± 1.2	6.7 ± 0.1
0.5	732.8 ± 17.5	51.4 ± 1.2	4.9 ± 0.1

## Data Availability

The data presented in this study are available on request from the corresponding author.
